# Metabolic profiling as a tool for prioritizing antimicrobial compounds

**DOI:** 10.1007/s10295-015-1666-x

**Published:** 2015-09-03

**Authors:** Changsheng Wu, Young Hae Choi, Gilles P. van Wezel

**Affiliations:** Molecular Biotechnology, Institute of Biology, Leiden University, Sylviusweg 72, 2333 BE Leiden, The Netherlands; Natural Products Laboratory, Institute of Biology, Leiden University, Sylviusweg 72, 2333 BE Leiden, The Netherlands; Department of Microbial Ecology, Netherlands Institute of Ecology (NIOO-KNAW), Wageningen, The Netherlands

**Keywords:** Metabolomics, NMR, Antibiotic production, *Streptomyces*, Antimicrobial drug resistance

## Abstract

Metabolomics is an analytical technique that allows scientists to globally profile low molecular weight metabolites between samples in a medium- or high-throughput environment. Different biological samples are statistically analyzed and correlated to a bioactivity of interest, highlighting differentially produced compounds as potential biomarkers. Here, we review NMR- and MS-based metabolomics as technologies to facilitate the identification of novel antimicrobial natural products from microbial sources. Approaches to elicit the production of poorly expressed (cryptic) molecules are thereby a key to allow statistical analysis of samples to identify bioactive markers, while connection of compounds to their biosynthetic gene cluster is a determining step in elucidating the biosynthetic pathway and allows downstream process optimization and upscaling. The review focuses on approaches built around NMR-based metabolomics, which enables efficient dereplication and guided fractionation of (antimicrobial) compounds.

## Introduction

The discovery and development of antibiotics to treat bacterial infections is one of the greatest triumphs of modern medicine. However, the exponential increase of antimicrobial resistance means that bacterial infections now once more pose a major threat to human health [126]. The high frequency of re-discovery of known molecules thereby frustrates screening efforts, and this necessitates new approaches to bolster the antibiotic pipelines [6, 23, 39, 95]. Filamentous fungi and bacteria of the order of *Actinomycetales* are the major producers of biomedical and agricultural natural products, and these microorganisms are responsible for producing the vast majority of the known antibiotics [9, 85]. Some two-thirds of all antibiotics are produced by actinomycetes, the majority of which are sourced by members of the genus *Streptomyces*. However, this is likely to represent only a tiny portion of the repertoire of total chemical space of bioactive compounds that these microorganisms may produce [9, 85]. Firstly, while the number of filamentous microbes in soil and marine environments is enormous, the bulk of them resist cultivation in the laboratory and thus escape screening and exploitation [53]. Recent work showed that enabling growth of these ‘uncultivable’ microorganisms might open up a new area of the chemical space of natural products [68, 129]. Secondly, many natural product biosynthetic gene clusters (BGCs) of cultivable microbes remain silent during standard laboratory cultivation [10, 136]. As an example, genome sequencing of actinomycetes revealed that the producing capacity of even the best-studied model organisms has been grossly underestimated [8, 27, 51, 119]. Thirdly, those gene clusters that are expressed, particularly if expression is relatively low as compared to other bioactive compounds produced by the same organism, often escape discovery due to the very demanding chemical analysis or chromatographic purification that is required [94]. New approaches are therefore required to exploit the huge unexplored reservoir of bioactive natural products and rejuvenate the drug-discovery pipelines.

The preferred method of identifying compounds in complex mixtures is metabolomics, which is a high-throughput analytical technique that offers a global analysis of the products of all cellular metabolic reactions, regardless of the reactions that lead to their production [96]. Spectroscopic techniques, and in particular nuclear magnetic resonance (NMR) and mass spectrometry (MS), are applied to chemically profile the metabolites that are produced by microorganisms. In connection with a wide range of chemometric methods, the metabolic differences among experimental groups are subsequently unraveled by comparison [38, 131]. In this review, we discuss ways to enforce fluctuations in the production of bioactive compounds, and the application of metabolomics to facilitate uncovering those compounds, whereby we zoom in on NMR-based metabolic profiling methods, which are less commonly applied in microbial drug discovery.

## Approaches to elicit antibiotic production

Genome mining of actinomycetes on average reveals the presence of 30–40 secondary metabolite BGCs per species [125]. Only a small number of these gene clusters has been matched to a natural product. A major problem is that many BGCs remain silent or poorly expressed under routine screening conditions. If we are to even start attempting to visualize these compounds by metabolomics approaches, their activation is the first essential step. Generic approaches to activate silent antibiotics to uncover the hidden chemical diversity include: (i) changing the growth media [14, 137]; (ii) inducing antibiotic resistance, e.g. to streptomycin [91] or rifampicin [37, 48, 90]; (iii) the addition of chemical elicitors, such as *N*-acetylglucosamine [98], γ-butyrolactones [50, 128], or histone deacetylase inhibitors [2]; and (iv) microbial cocultivation [11, 49, 109]. Instead of inducing the production of an antibiotic, an interesting alternative may be offered by screening for inducible resistance, in particular associated with classes of antibiotics that are less frequently identified in classical screens. Growth on media containing the glycopeptide vancomycin was used to select actinomycetes that produce (and are therefore resistant to) glycopeptide antibiotics. Subsequent phylogeny-based screening based on specific biosynthetic genes led to the identification of the novel glycopeptide antibiotic pekiskomycin [114].

In terms of the activation of antibiotic production, a concept study was recently done using a collection of some 800 strains that were grown under 40 different growth conditions and then assayed for changes in the production of antimicrobial activity against the so-called ESKAPE pathogens [137]. This showed that many strains that fail to produce antibiotics under routine growth conditions, produce bioactivities that inhibit the growth of MDR pathogens under specific nutritional conditions or after addition of elicitors [137].

### Harnessing the regulatory mechanisms that control antibiotic biosynthesis

To develop new directed approaches, it is imperative that we better understand the underlying regulatory networks. The regulation of antibiotic production involves multiple regulatory cascades and networks. Here, we will guide the reader with some general principles gleaned from the model organism *Streptomyces coelicolor*; for more extensive reviews on the control of antibiotic production we refer the reader elsewhere [13, 69, 100, 120].

Antibiotic BGCs specify biosynthesis and modification, resistance, transport and pathway-specific control. The best-studied example of cluster-encoded, pathway-specific activators is that of the *Streptomyces* antibiotic regulatory proteins (SARPs). SARPs typically bind to direct repeats in promoter regions of biosynthetic genes, thereby activating transcription of some or all of the antibiotic biosynthetic genes in the cluster [127]. In *S. coelicolor*, the pathway-specific regulatory genes *act*II-ORF4, *cdaR* and *redD* encode the SARPs for actinorhodin (ACT), calcium-dependent antibiotic (CDA) and undecylprodigiosin (RED) production, respectively. Of these, *actII*-ORF4 and *redD* have been extensively studied. Deletion of these genes abolishes the production of actinorhodin or undecylprodigiosin, respectively [36], and their transcription is activated in a growth phase-dependent manner [4, 113]. Interestingly, positioning *redD* behind a developmental or nitrogen-responsive promoter is sufficient to produce undecylprodigiosin in aerial hyphae or bring it under nitrogen control, respectively, which suggests a lack of downstream control [122]. Hence, one effective approach to activate gene clusters is overexpression of their activator genes, but this involves genetic manipulation which is not amenable to high-throughput approaches.

Recent years have underlined the importance of global regulators as a higher order regulatory network, and their possible application for the activation of antibiotic production. Many pleiotropic regulators characterized so far are required only under specific environmental conditions [12, 121]. The nutrient sensory GntR-family regulator DasR controls among others aminosugar metabolism and transport, the chitinolytic system and antibiotic production [21, 22, 26, 97, 98, 112]. DasR is a highly pleiotropic regulator, as demonstrated by recent systems biology analysis of chitin- or *N*-acetylglucosamine-induced cultures of *S. coelicolor* [84, 110]. DasR directly controls the transcription of *act*II-ORF4 and *redD* as well as *redZ*, a response regulator required for the activation of *redD* and thus of undecylprodigiosin production [98]. Systems-wide DNA binding experiments using ChIP-chip analysis revealed that in *S. coelicolor* DasR likely controls all pathway-specific activator genes [110], but this appears to be more an exception than a rule. The activity of DasR is modulated by metabolic derivatives of *N*-acetylglucosamine, and addition of this amino sugar to the culture media activates antibiotic production in several actinomycetes, including cryptic antibiotics [98].

Interesting cross-talk is seen for DasR with other higher order antibiotic regulators. One is the TetR-family regulatory protein AtrA that in *S. coelicolor* is required for the transcription of *actII*-ORF4 [118] and in *S. griseus* of *strR*, the pathway-specific activator gene of streptomycin production [47]. The antibiotic activating role of AtrA may be widespread, as suggested by recent data that AtrA also controls biosynthesis of the important lipoglycopeptide antibiotic daptomycin in *Streptomyces roseosporus* [76]. AtrA is controlled by the level of phosphate and repressed by the PhoRP system [101]. AtrA activates development [60] as well as *N*-acetylglucosamine import [88], and thus antagonizes the repressing activities of DasR, whereby the metabolic balance likely plays a deciding role on the net outcome. Further complexity is offered by Rok7B7, a member of the ROK family of proteins, which are predominantly sugar regulatory proteins and sugar kinases, including glucose kinase [73, 115]. Rok7B7 also pleiotropically affects primary and secondary metabolism, and is like AtrA required for actinorhodin production [79, 111]. Recent evidence suggests that Rok7B7 may be activated by a derivative of the C5 sugar xylose [111].

Other pleiotropic antibiotic regulators involved in the control of *actII*-ORF4 are the large SARP-family regulator AfsR and the phosphate regulator PhoP. AfsR derives its name from a putative relationship to synthesis of the hormone-like signaling molecule A-factor, belonging to the γ-butyrolactones. The A-factor-responsive AdpA controls the onset of development and antibiotic production in *S. griseus* [46, 116], but the precise relationship with AfsR (if any) so far remains unresolved. In *S. coelicolor*, AfsR is conditionally required for Act and Red production [36] and its control is somehow transmitted through control of the small downstream gene *afsS*, thereby activating its transcription [67]. While the precise function of *afsS* is unclear, similarly to AtrA, its pleiotropic and positive effect on antibiotic production makes it an attractive target for the activation of antibiotic production. PhoP represses actinorhodin production in response to phosphate [106], and it may also relay its control via *afsS* [101] These are examples to highlight the complexity of the control of antibiotic production, whereby an astonishing number of around 15 regulatory proteins have been shown to control *act*II-ORF4 alone [121]. Improved understanding of these multiple and intertwined regulatory networks will allow scientists to design new approaches to activate poorly expressed BGCs.

### Connecting eliciting approaches to metabolomics-driven lead discovery

In the framework of this review, the activation of gene expression is important to activate silent gene clusters and particularly to achieve significant fluctuation of the production of (cryptic) antibiotics and thus differential bioactivity between cultures; this serves as enabling technology for statistical correlation between a bioactivity of interest and the responsible (sought-after) compounds. Having established such differential expression, the next challenge is to efficiently identify the induced antimicrobial compounds against a background of an inevitably highly complex metabolome matrix.

**Fig. 1:**
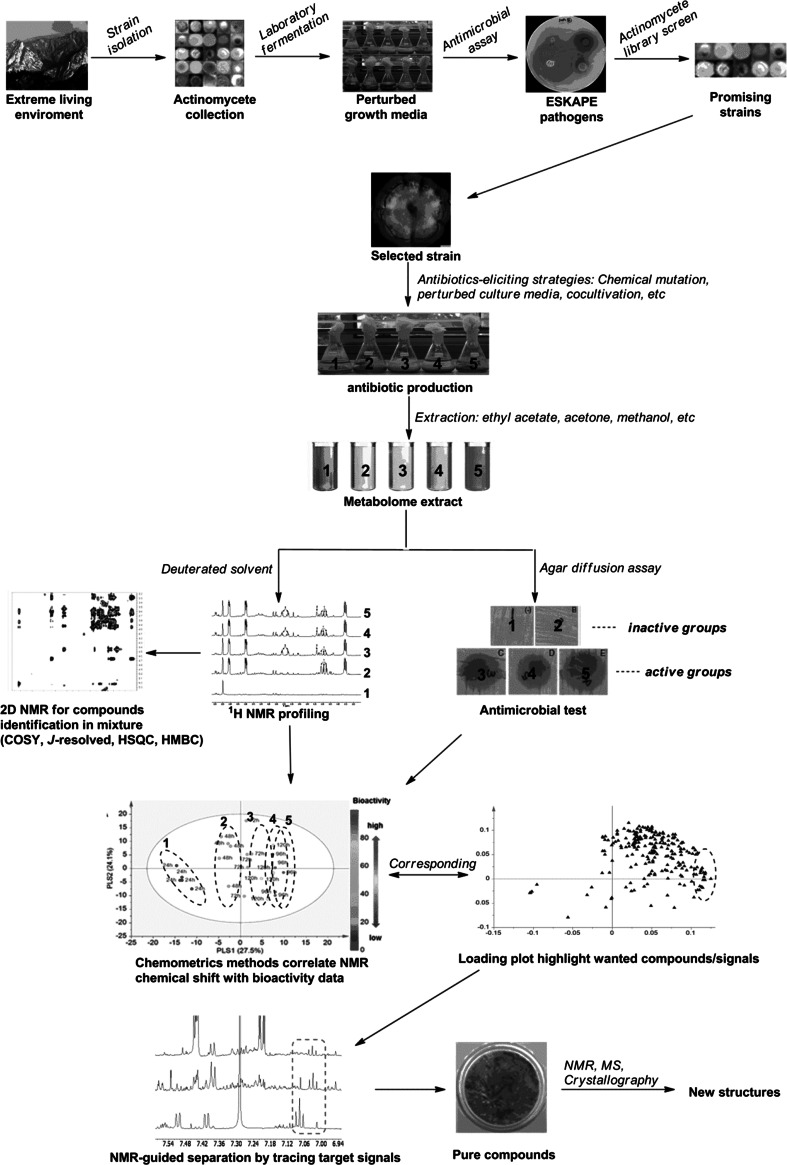
A pipeline for the discovery of antibiotics produced by actinomycetes based on NMR-based metabolomics. Strains are subjected to eliciting approaches to activate the production of poorly expressed antimicrobials, followed by NMR-based metabolomics to identify the bioactive molecules. ^1^H NMR profiling of active and inactive groups, aided by 2D NMR, allows dereplication of known molecules and chemometric methods then correlate proton signals in the active fractions to the bioactive compound(s)

Metabolomics is an effective tool for facilitating the discovery of new antibiotics, because it allows the multivariate comparison of active and inactive metabolic samples, highlighting particularly the differentially produced compounds, which serve as potential biomarkers, thereby avoiding chemical redundancy in the very early stage [59, 63, 93]. Integrating metabolomics approaches with new eliciting strategies should allow scientists to streamline their drug-discovery pipeline, so as to prioritize novel molecules over repeated (and frustrating) isolation of known molecules. A schematic representation of a feasible antibiotics discovery pipeline based on NMR-based metabolomics is presented in Fig. 1. Compounds can be readily identified in complex biological matrices without time-consuming chromatographic separation, further aided by 2D NMR experiments. In the next section, we detail some of the NMR techniques that are most appropriate for metabolomics approaches.

## Metabolomics of natural products based on NMR spectroscopy

NMR techniques that are most frequently used in NMR metabolomics are ^1^*J*-resolved, COSY, TOCSY, HSQC, and HMBC [58, 59, 74, 89]. ^1^H NMR signals corresponding to molecules that exist in both the active and the inactive groups are discarded based on lack of statistical relevance [105]. Chemometric analysis is then used for further data mining of the ^1^H NMR data, extracting information that is intractable to visual detection. In principle, the methodology of NMR measurements, spectral data preprocessing, and multivariate data analysis basically follow a protocol that was originally designed for analysis of plant metabolites [58]. The ^1^H NMR signals correlated with the better bioactivity and separation of the active from the inactive groups are used as probing tools for NMR-guided isolation of the sought-after bioactivity. Advantages of NMR-based over MS-based (LC–MS or GC–MS) metabolomics are: (i) the chemical shifts and splitting patterns of proton resonance data provide partial structural information that allows early assessment of the type of compound causing the bioactivity, thus aiding in avoiding redundancy; (ii) the analysis of crude mixtures can be done in very short time (in the order of minutes), with high signal robustness (i.e. minimum technical variation), so as to guide scientists in a medium- to high-throughput environment. These advantages should be weighed against the lack of true high-throughput application, which is a key selling point of MS-based approaches. As soon as the major interfering signals are removed from the NMR spectra, the material (even if not yet pure) is likely of sufficient quality for full structure elucidation. In some cases, structural elucidation of unknown compounds is challenging, especially for those entities with unprecedented molecular scaffold. Two-dimensional NMR (2D NMR) techniques have formed the foundation of most contemporary approaches for structure elucidation [16], allowing assembly of the molecular architecture by defining atom-to-atom connectivity. In general, NMR-based metabolomics uses a combination of 2D NMR techniques for deconvoluting metabolites in congested mixtures, namely *J*-resolved, ^1^H-based 2D (COSY and TOCSY), ^13^C-based 2D NMR (HSQC and HMBC) [16]. *J*-resolved solves the signal purity of ^1^H NMR by showing splitting pattern with coupling constants in F2 axis which requires for chemical structures of corresponding signals. COSY and TOCSY give information of ^1^H connectivity within the same molecule. Along with the connectivity, the ^1^H-based COSY and TOCSY also help signal identification in overlapped regions. Many signals are overlapped which obscures some of the peaks in the mixture, but these can be deconvoluted by the correlations in the 2D NMR spectra. ^13^C-related spectra such as HSQC and HMBC give more detailed structural information, particularly for the carbon skeleton. Although important for identifying the carbon skeleton of organic compounds, ^13^C-NMR is rarely applied in NMR-based metabolomics because of the low abundance of ^13^C in nature and its long relaxation time, and also because ^13^C broad-band decoupling cannot be used quantitatively. HSQC and HMBC are therefore primarily applied to support the ^1^H NMR data.

**Fig. 2:**
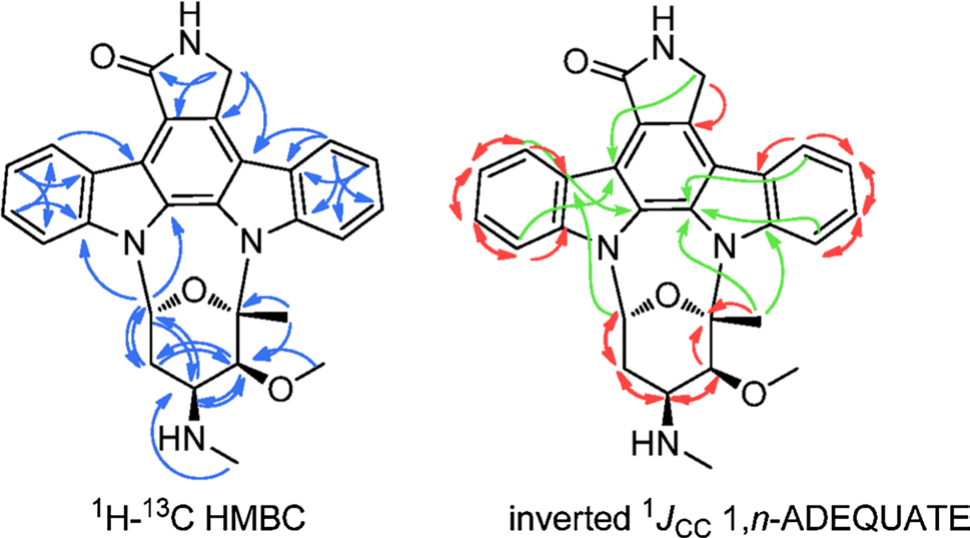
Diagram of HMBC (*blue anticlockwise semi-circle arrows*), and inverted ^1^ *J*_CC_ 1,* n*-ADEQUATE correlations by using the proton-deficient alkaloid staurosporine as an example. The skeleton of this model compound can be unequivocally assigned by HMBC + inverted ^1^ *J*_CC_ 1,* n*-ADEQUATE strategy. In inverted ^1^ *J*_CC_ 1,* n*-ADEQUATE, ^1^ *J*_CC_ correlations are shown as *red clockwise semi-circle arrows*, while important ^3^ *J*_CC_ correlations are shown as *green clockwise semi-circle arrows*

Deciphering the structure of organic molecules by using conventional routine suites, based on for example *J*-resolved-COSY (or TOCSY), HSQC and HMBC [87] often gives rise to misassignment. This partly arises from the inherent challenges of differentiating two-bond heteronuclear (H ⇢ C, ^2^*J*_CH_) correlations from three-bond correlations (^3^*J*_CH_) in the HMBC spectrum. In addition, as HMBC normally just provides ^2^*J*_CH_ and ^3^*J*_CH_ but rarely ^4^*J*_CH_ correlations, structure elucidation becomes progressively challenging the more molecules become proton-deficient [104]. To address these problems, many other approaches have been tried such as combined HSQC-TOCSY [61]. Researchers from Merck proposed that adequate sensitivity double-quantum (1, *n*-ADEQUATE) NMR spectroscopy should be reconsidered as a complement for HMBC in structure elucidation [77]. 1,1-ADEQUATE exclusively presents ^1^*J*_CC_ correlations and can help distinguish ^2^*J*_CH_ from ^3^*J*_CH_ correlations in the HMBC spectrum, which would be a powerful method for determining the proton-rich small molecules by using a combination of 1,1-ADEQUATE + HMBC [19, 83]. 1, *n*-ADEQUATE experiments predominantly yield homonuclear ^3^*J*_CC_ correlations (equal to ^4^*J*_CH_) that are difficult to obtain with HMBC NMR. This feature makes 1,* n*-ADEQUATE a complement for HMBC in defining proton-deficient structures, such as a large group of aromatic polyketides (PKS) produced by actinomycetes [17, 83]. Furthermore, newly developed inverted ^1^*J*_CC_ 1,* n*-ADEQUATE spectroscopy allows the integration of 1,1-ADEQUATE and 1,* n*-ADEQUATE information in a single spectrum, while they remain distinguished by plotting the output in different colors (Fig. 2) [78].

Though NMR spectroscopy is undoubtedly powerful in the structure elucidation, it works better on pure organic compounds, as otherwise signal-overlapping issues come into play. To address this, chromatography-hyphenated NMR (HPLC-NMR) has been introduced into metabolic analysis [130]. The major advantages of on-flow HPLC-NMR lie in the real-time access to structure information of chromatographic peaks by using NMR spectrometers as detector [75]. To balance the inherent lower sensitivity of NMR spectra and the limited injection volume onto the HPLC unit, solid-phase-extraction (SPE) is coupled to trap the analytes onto an identical SPE cartridge when repeated HPLC analysis, followed by final elution with a deuterated solvent into miniaturization tube for NMR analysis [103]. By using this LC-SPE-NMR approach [64, 108], comprehensive structural information about chemical composition of crude extracts can be obtained online, thus enabling early and prospective assessment of the valuable components of an extract or fraction [52]. Furthermore, when integrating MS into the system, like LC-DAD-SPE-MS-NMR instrumentation [33, 70], the architecture of novel compounds can be elucidated *de novo* and online without laborious purification of the analyte(s) from its highly complex matrix.

## Connecting genotype to chemotype: integrating genomics with metabolomics

Before looking at applications of metabolomics to identify novel compounds, in this section we will look into how metabolomics may aid in connecting a natural product of interest to its BGC. This is a crucial step as it facilitates molecular approaches such as directed mutagenesis, overexpression of pathway-specific regulators and heterologous expression. Furthermore, identification of the gene cluster is often required for determining the precise biosynthetic pathway.

Next-generation sequencing (NGS) technologies have uncovered the genetic architecture of thousands of BGCs, and this has revolutionized the drug-discovery approaches [24]. Exploiting this rapidly increasing source of information, a recent network analysis of the phylogeny and distribution of BGCs in microbial genomes revealed a vast number of yet underexplored genetic resources, belonging to many hundreds of gene cluster families [20].

Gene clusters such as those for PKS or nonribosomal peptides (NRPS) are readily identified using bioinformatics, and to some extent the domain structures of the biosynthetic proteins allows prediction of the molecule that they specify [5, 66]. In recent years, excellent bioinformatics packages have been developed for the identification of BGCs. These include antiSMASH (ANTIbiotics & Secondary Metabolite Analysis SHell [81] and SMURF (Secondary Metabolite Unknown Regions Finder), as well as packages that enable the identification of biosynthesis genes for specific subclasses of NPs (reviewed in [34] ). Examples are SBSPKS (Structure Based Sequence Analysis of Polyketide Synthases) for polyketide BGCs [3], NRPSPredictor for NRPS gene clusters [99] and BAGEL for the identification of biosynthetic clusters for bacteriocins and lantibiotics [28].

### From genotype to chemotype: bioinformatics-guided natural product discovery

**Fig. 3:**
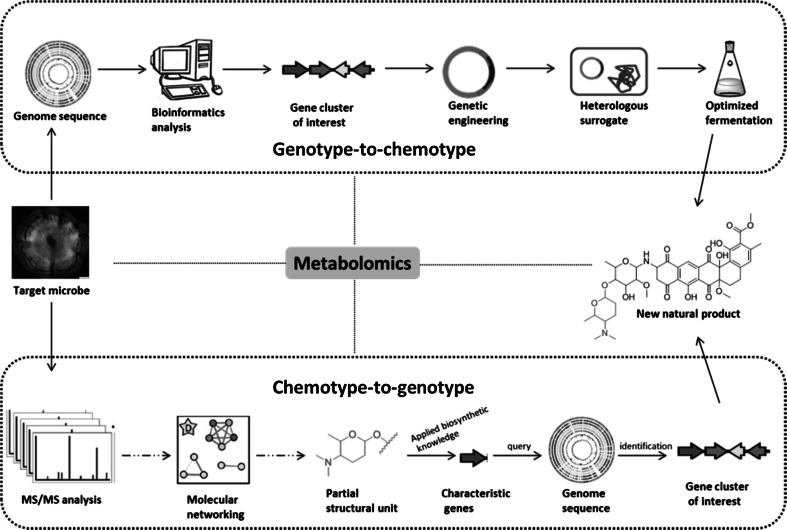
General workflows for genotype-to-chemotype (**a**) and chemotype-to-genotype (**b**) genome mining for natural product discovery

How can we determine whether the compound that is derived from a given BGC is novel (*genotype to chemotype*) or, conversely, link a natural product of interest to its gene cluster (*chemotype to genotype*)? The *genotype*-*to*-*chemotype* roadmap (Fig. 3a) [56, 57] provides a glimpse of ‘hidden treasures’, e.g. aided by the exploitation of pathway-specific regulators [40, 65] or via heterologous gene expression [7, 92]. Metagenomic libraries from environmental samples thereby allow scientists access to the ‘dark matter’, namely the vast biosynthetic potential of uncultivable microbes [15, 54]. However, an intrinsic disadvantage of genome-based bioprospecting is that current technologies target a single BGC at the time, which is time-consuming, while the discovery efficiency is low as it is very difficult to prioritize ‘promising’ gene clusters. After all, if a gene cluster appears novel, it does not mean that the compound has not already been identified in HT screening regimes in the pre-genomic era. We predict, however, that synthetic biology and heterologous expression of gene clusters will soon become part of a more automated environment, which would make these approaches much more feasible. In addition, approaches directed at the (heterologous) expression of gene clusters depend on the functional analysis of biosynthetic enzymes and/or detection of intact clusters, while biosynthetic reactions may be catalyzed by enzymes that are not encoded by the gene cluster itself and/or by enzymes with low specificity [102], or are catalyzed non-enzymatically [80, 132]. Indeed, metabolites are the end products of many cellular processes, so that the bioactive end product is not necessarily specified by individual genes or clusters [96].

In the case of intertwined metabolic and biosynthetic pathways giving rise to a single compound, targeted genomic approaches lose their effectiveness [117, 124, 132]. In such cases, metabolomic approaches are required to bridge the gap between bioinformatics-driven gene cluster analysis and NP discovery [30, 62]. The fundamental principle is based on the fact that structurally related NPs are typically characterized by similar MS/MS fragmentation patterns. The MS/MS structural relatedness among molecules can be detected in an automated manner, and can subsequently generate a molecular network wherein analogues cluster together. In this way, known structures and “known unknowns” can be identified in extracts, offering an effective dereplication strategy and a way of prioritizing novel compounds [134].

### From chemotype to genotype: metabolomics to identify biosynthetic gene clusters

MS/MS analysis allows the detection of “structural unit tags” or building blocks whose biosynthesis needs specific enzymes bearing substrate specificity. Candidate genes can be used as query against the genome databases to locate the target gene cluster for the compound(s) of interest. MS-based metabolomics in conjunction with molecular networking builds up a *chemotype*-*to*-*genotype* roadmap (Fig. 3b) [44, 56, 57, 62] for NP discovery, directly linking secondary metabolites to their corresponding gene clusters. Examples for this concept include peptidogenomics [56, 86], glycogenomics [57] and natural product proteomining [43]. Peptidogenomics is an MS-based genome-mining method that links peptide natural products to their BGCs through iteratively matching tandem MS (MS^n^) sequence tagging with genomics-derived peptide structures [56], supported by software automation packages such as the recently developed Pep2Path [82]. For an excellent review of peptidogenomics techniques and their application we refer the reader elsewhere [62]. Glycogenomics is a *chemotype*-*to*-*genotype* approach for fast characterization of glycosylated natural products (GNPs) and their biosynthetic pathways. Tandem mass spectrometry (MS^n^) allows rapid identification of the structural information of sugar substituents of GNPs in microbial metabolic extracts [32, 45]. The biosynthesis of certain sugars need specific enzymes; as an example, genes for forosamine production encode 2,3- and 3,4-dehydratases, 3-ketoreductase, aminotransferase and *N*,*N*-dimethyltransferase. Applying the bacterial sugar biosynthetic knowledge, MS^n^-characterized glycosyl groups can be matched to corresponding glycosylation genes. In microbial genomes, the glycosylation-associated biosynthetic genes are usually co-clustered with those for a wide range of aglycones. Programs such as antiSMASH are specifically designed with the identification of such signature BGCs in mind. The relevant aglycone biosynthetic genes are then identified, which in turn guides further isolation and structural identification of glycoconjugates by applying knowledge of the aglycone-specific BGC. As aglycones could be any type of structural scaffolds, for sugar-modified NPs, glycogenomics has an advantage over peptidogenomics. This technique therefore expands the applicability of MS-guided genome mining for different classes of NPs, such as nonribosomal and ribosomal peptides, PKS, terpenes, and alkaloids [32]. Furthermore, since glycosylated products are produced via the decoration of an aglycone, which is often bioactive by itself, we envisage that glycogenomics can be extended to discover even non-glycosylated NPs that serve as intermediates for final GNPs.

Natural product proteomining is based on the concept that gene cluster expression profiles correlate directly to the level of the corresponding natural product [43]. When growth conditions are chosen such that a bioactivity of interest is differentially produced, e.g. high under some conditions and intermediate or not at all in others, statistical correlation between bioactivity (metabolomics) and gene expression profiles (proteomics, RNAseq) will allow prioritizing those gene clusters with the optimal match. Since the metabolomics will also provide clues on the type of molecule, evidence of the BGC responsible for the product of interest can thus be obtained, with the predictive value depending on the number of candidate BGCs present in the genome and the number of samples analyzed [43]. The concept of metabolomics aiding genomics in finding new NPs is also exemplified by the case of myxoprincomide from the *Myxococcus xanthus* [25]. Initially, 13 BGCs in *M. xanthus* DK1622 were found for which the metabolites remained elusive. After targeted inactivation of these cryptic gene clusters, MS-based metabolomics was applied to pairwise compare extracts of mutants and the wild-type strain, highlighting the subtle metabolic differences by statistical filtering. Thus, a metabolite with molecular formula C_45_H_76_N_10_O_16_ was assigned to a NRPS/PKS hybrid gene cluster and identified as a molecule with the unprecedented structural skeleton of myxoprincomide. Gene knockout/untargeted metabolomics strategy could also facilitate discovery of intermediates and/or new side products of biosynthesis pathway, as exemplified by characterization of 2-alkyl-4,5-dihydrothiazole-4-carboxylates from *Pseudomonas aeruginosa* [55, 123]. Comparison of the extracellular metabolome of *P. aeruginosapch* mutants with that of the wild-type strain identified 198 secondary metabolites regulated by these pathway-specific *pch* genes. Therefore, perturbation of the expression of global or pathway-specific regulators followed by metabolomics is a promising strategy to rapidly connect molecules to genes or gene clusters.

## Experimental approaches based on NMR metabolomics

**Fig. 4:**
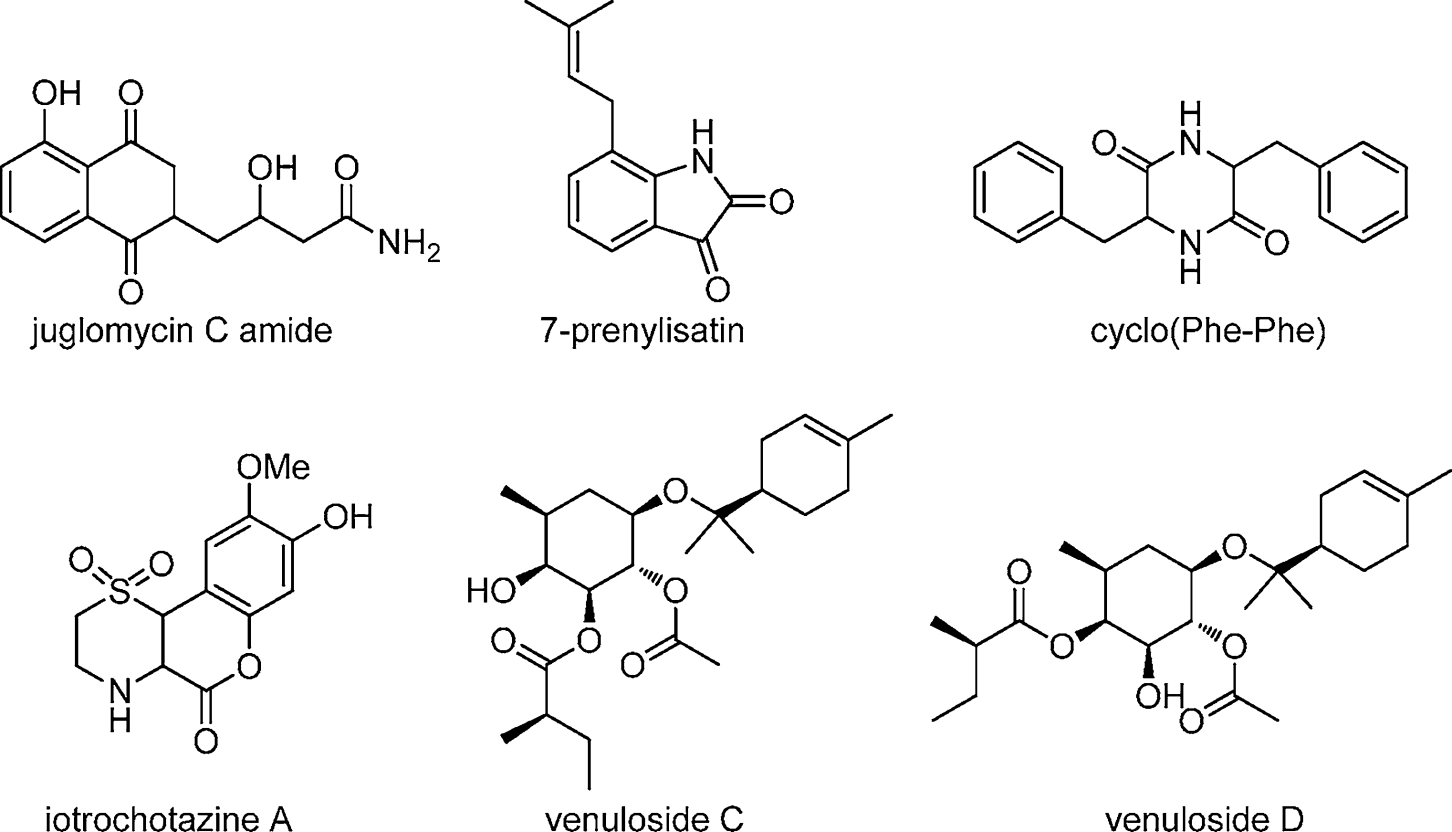
Chemical structures of the compounds mentioned in the final section of the review (“Experimental approaches based on NMR metabolomics”)

As explained above, metabolomics approaches allow elucidating the nature of poorly bioactive molecules in complex mixtures, and for this achieving differential production of the bioactivity of interest is a key step. Such fluctuation may be achieved by among others different culturing conditions, inducing drug-resistance or by co-culturing [38, 131]. NMR-based metabolomics is very common for identifying plant NPs, but has yet seen relatively little application in microbial drug discovery. A general scheme is presented in Fig. 1. We recently applied NMR-based metabolomics for the mining of novel soil isolates with promising antibiotic-producing potential, of which we provide a few examples here for illustration purposes. To identify a bioactivity produced by the soil isolate *Streptomyces* sp. MBT70, the strain was grown in media supplemented with different additives followed by ^1^H NMR spectroscopy and statistical comparison of the extracts by partial least square modeling-discriminant analysis (PLS-DA). A main discriminator between bioactive and inactive cultures was a signature H-5 residue belonging to a naphthoquinone. Further HPLC fractionation targeting naphthoquinones then led to the isolation and identification of juglomycin C amide (Fig. 4) [43]. The generation of streptomycin resistant (Str^R^) mutants in soil isolate *Streptomyces* sp. MBT28 resulted in the specific activation of a bioactive compound, which was subsequently identified by comparing the active (Str^R^-induced) and non-active fractions by ^1^H NMR and statistical analysis by projection to latent structures (PLS); this allowed correlation of the enhanced bioactivity to a set of distinctive aromatic signals. NMR-guided separation by tracking characteristic ^1^H NMR signals as probes, resulted in the characterization of the isatin-type antibiotic 7-prenylisatin (our unpublished data). As a third example, co-cultivation of the filamentous model microbes *S. coelicolor* and *Aspergillus niger* substantially influenced their NPs profiles. NMR spectroscopy of extracts obtained from the separate strains and the coculture combined with multivariate data analysis revealed diketopiperazine-type compounds like cyclo(Phe-Phe) (Fig. 4), which were exclusively produced by *A. niger* when grown in co-culture with *S. coelicolor* [133].

^1^H NMR based profiling also finds its application in lead discovery. Quinn and coworkers integrated solid phase extraction (SPE) with NMR fingerprinting. An extract library was prepared featuring lead-like enhanced (LLE) fractions selected on the basis of favorable physicochemical properties [18], and this LLE library was subsequently profiled by ^1^H NMR spectroscopy to present the structural information of the small molecules contained in the library. In this way, the complicated and overlapping ^1^H NMR spectra typical of crude extracts were avoided by prefractionation, facilitating the detection of minor compounds. Using the proton signals as tracking signals for NMR-guided isolation, an unprecedented scaffold of iotrochotazine A was identified in LLE-fractionated extracts from sponges [41] and low-abundant venulosides C and D (Fig. 4) in extracts from plants [42].

**Fig. 5:**
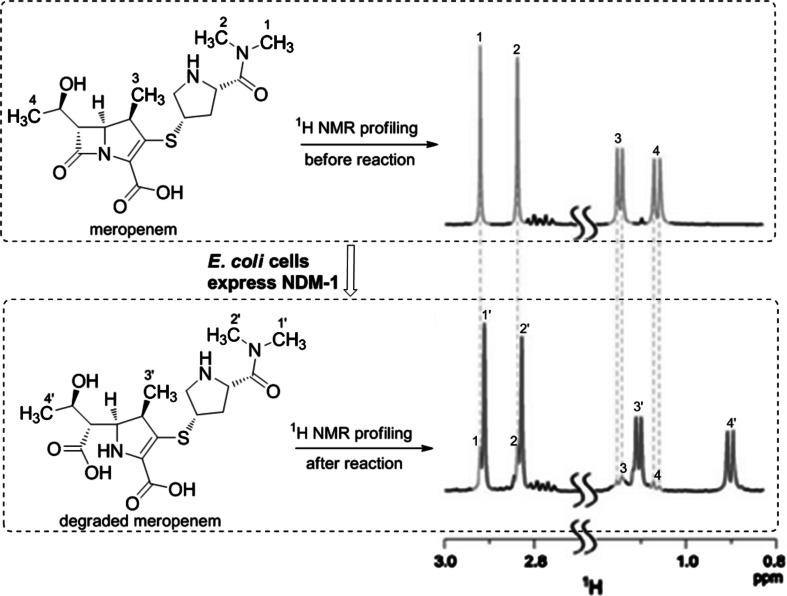
In vivo ^1^H NMR spectroscopy monitors NDM-1 activity in living *Escherichia coli* cells. Cyclic amide ring in β-lactam antibiotic meropenem is hydrolyzed under catalysis of enzyme NDM-1. The ^1^H NMR chemical shifts of four characteristic methyls (*green*) in the substrate accordingly move upfield in the hydrolytic product (*red*). This reaction can be monitored in real-time without disturbing the experimental system

An important class of NPs in the fight against antibiotic resistance is that of resistance inhibitors. Penicillins are still the most administered antibiotics [29, 107], but resistance caused by β-lactamases was discovered almost as soon as the antibiotic itself [1] and has become a major problem for their clinical application [126]. The combined use of β-lactam antibiotics together with β-lactamase inhibitors is exemplified by the combination of amoxicillin and clavulanic acid, marketed as Augmentin^®^ [31, 35]. A target-based in-cell NMR approach was recently proposed for use in high-throughput screening approaches to identify resistance inhibitors with activity against a specific β-lactamase [71]. The New Delhi metallo-β-lactamase (NDM-1), which opens the cyclic amide ring in β-lactams antibiotics [135], was monitored in real time in *E. coli* cells by ^1^H NMR spectroscopy [72]. The approach is summarized in Fig. 5. The chemical shifts of four characteristic methyl groups are different between the substrate meropenem and its NDM-1-degraded product, which was used as a reporter of the lytic reaction. Inhibition of NMD-1 enzymatic activity was readily detected since the characteristic methyl groups of meropenem remain unshifted in the ^1^H NMR spectrum. NMR-guided fractionation was then applied to trace the responsible antibiotic adjuvants in the active fractions.

## Conclusions

In a time where dereplication is one of the major challenges in screening efforts, metabolomics offers an effective strategy to prioritize novel molecules produced by microorganisms. Combination of eliciting strategies with comprehensive chemometric comparison of secondary metabolomes and genomics is an excellent alternative over traditional HT approaches. Metabolomics thereby complements (meta-)genomics and synthetic biology approaches in the mining of producing organisms, in particular for poorly expressed (cryptic) molecules. In addition, connecting chemotype to genotype and vice versa is important to facilitate molecular approaches and to accelerate unraveling of the biosynthetic pathway, and thus enable metabolic engineering and upscaling. Also taking the huge promise of the unculturable microorganisms into account, it is clear that natural product discovery is far from an echo from the past. Instead, as noted by many scientists worldwide, we may well be witnessing a new era of drug discovery, where many different disciplines such as genomics, metabolomics, bioinformatics, synthetic biology, ecology, chemical biology and industrial screening come together to meet the drug-discovery challenges and deliver new solutions to treat infectious diseases associated with drug resistance.
